# Personal Authentication Analysis Using Finger-Vein Patterns in Patients with Connective Tissue Diseases—Possible Association with Vascular Disease and Seasonal Change -

**DOI:** 10.1371/journal.pone.0144952

**Published:** 2015-12-23

**Authors:** Miyuki Kono, Naoto Miura, Takao Fujii, Koichiro Ohmura, Hajime Yoshifuji, Naoichiro Yukawa, Yoshitaka Imura, Ran Nakashima, Takaharu Ikeda, Shin-ichiro Umemura, Takafumi Miyatake, Tsuneyo Mimori

**Affiliations:** 1 Central Research Laboratory, Hitachi Ltd., Kokubunji-shi, Tokyo, Japan; 2 Department of Rheumatology and Clinical Immunology, Kyoto University Graduate School of Medicine, Kyoto, Japan; 3 Department of Dermatology, Wakayama Medical University, Wakayama, Japan; 4 Graduate School of Biomedical Engineering, Tohoku University, Sendai-shi, Miyagi, Japan; Nippon Medical School Graduate School of Medicine, JAPAN

## Abstract

**Objective:**

To examine how connective tissue diseases affect finger-vein pattern authentication.

**Methods:**

The finger-vein patterns of 68 patients with connective tissue diseases and 24 healthy volunteers were acquired. Captured as CCD (charge-coupled device) images by transmitting near-infrared light through fingers, they were followed up in once in each season for one year. The similarity of the follow-up patterns and the initial one was evaluated in terms of their normalized cross-correlation *C*.

**Results:**

The mean *C* values calculated for patients tended to be lower than those calculated for healthy volunteers. In midwinter (February in Japan) they showed statistically significant reduction both as compared with patients in other seasons and as compared with season-matched healthy controls, whereas the values calculated for healthy controls showed no significant seasonal changes. Values calculated for patients with systemic sclerosis (SSc) or mixed connective tissue disease (MCTD) showed major reductions in November and, especially, February. Patients with rheumatoid arthritis (RA) and patients with dermatomyositis or polymyositis (DM/PM) did not show statistically significant seasonal changes in *C* values.

**Conclusions:**

Finger-vein patterns can be used throughout the year to identify patients with connective tissue diseases, but some attention is needed for patients with advanced disease such as SSc.

## Introduction

There are many kinds of biometric authentication techniques, such as fingerprint authentication [1–4], iris recognition [5–6], face recognition [7–9], and hand geometry [10,11] and hand vein pattern authentication [12,13]. Biometrics techniques have come to be used for access control in automated teller banking (i.e., at ATMs), PC log-on procedures, and so forth. And there is international standardization activity devoted to biometric technologies [14].

Hitachi Central Research Laboratory had since 1997 been studying personal identification techniques based on finger-vein patterns obtained using near-infrared (IR) light. Light transmission through tissues is greatest within the red and near-IR wavelength bands [15–17]. At visible wavelengths, transmission decreases because of the electric absorption in tissue pigments, such as hemoglobin, myoglobin, and melanins. At longer IR wavelengths, the fundamental vibration modes of water bonds cause broad and intense absorption. At near-IR wavelengths, hemoglobin has lower absorbance than at visible wavelengths, but its absorbance is higher than that of other tissue proteins. Incoming light is absorbed to tissue pigments or scattered around the bone in the finger. Acquired image by outgoing light from the finger has only an information around the skin. Arteries locates deeper in the body and veins locates closer to the skin in general. As a result of that, we can acquire an image of the finger-vein pattern by using near-IR light.

The possibility of a personal identification technique using finger-vein patterns acquired by transmitting light through a hand was indicated about twenty years ago in Japan [18], but the technique had not been demonstrated. We have established and improved the technique [19–21], and it has already been commercialized [22, 23]. Our feasibility study in 2000 had been done on healthy volunteers as the subjects [19], thus we still had to study how this identification system works with people who have diseases that affect the blood flow in peripheral tissues. Connective tissue diseases are of particular interest in this regard because people with these diseases often show vascular disturbance of the fingers, such as Raynaud’s phenomenon.

In this study we have tried to examine how the connective tissue diseases affect the identification of finger-vein patterns. For this purpose, we have acquired the finger-vein pattern images of patients with connective tissue diseases and examined whether their patterns have any characteristics differentiating them from those of healthy volunteers and whether there are any problems with using our finger-vein identification technique on people with connective tissue diseases.

## Materials and Methods

### Experimental apparatus

**Fig 1:**
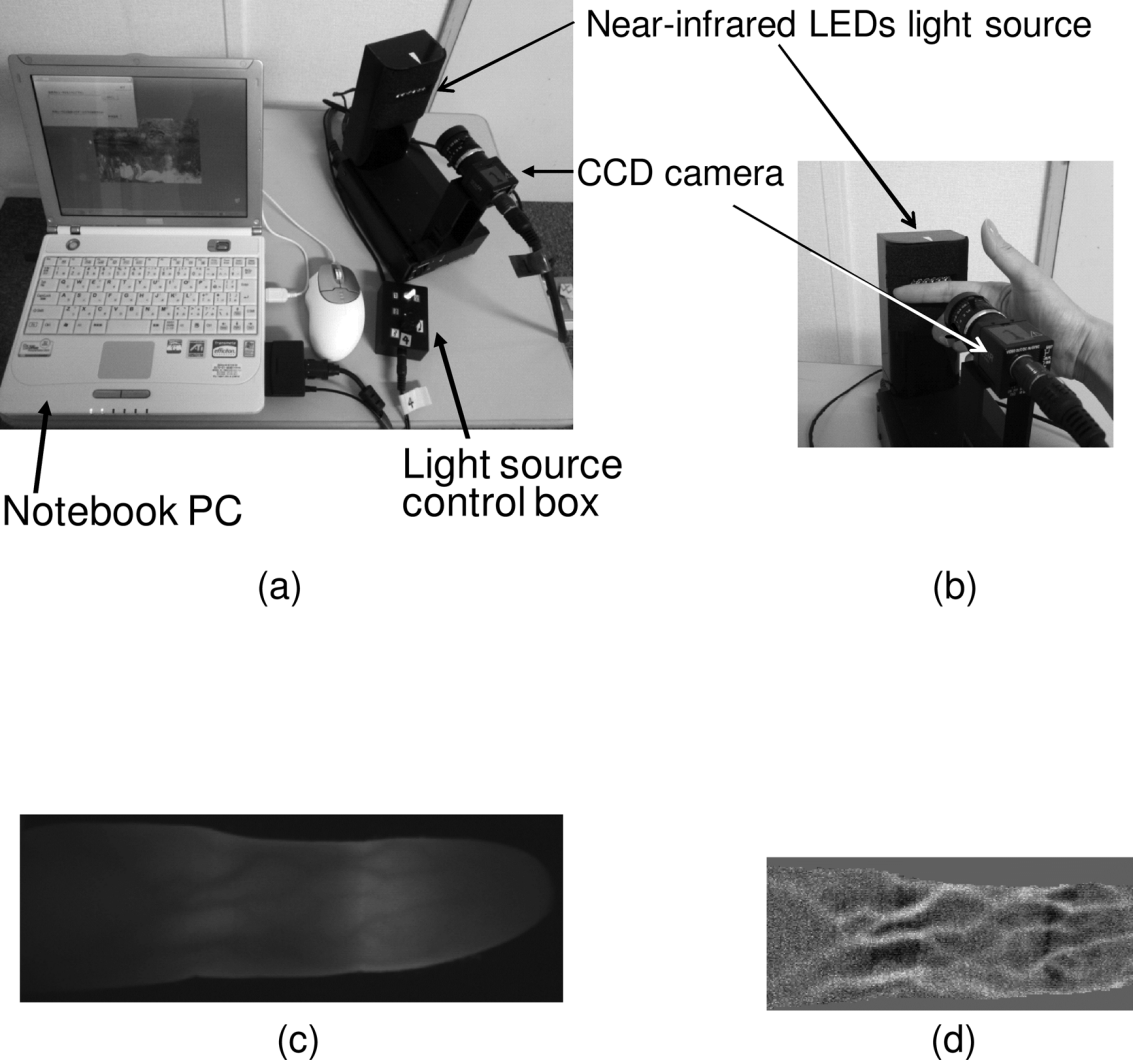
Instruments of finger-vein pattern authentication. (A) Experimental set-up for capturing finger-vein pattern images is composed of a near-infrared light source, a CCD camera, a video capture card, and a laptop computer. (B) A finger put against the light source to capture its vein pattern image. (C) Example of an acquired finger-vein pattern image. (D) A processed image.

Fig 1A shows the experimental set-up used to capture finger-vein patterns and shows the appearance of the light source. An array of mold type near-IR light emitting diodes (LEDs) (L890-06AU, Epitex, Kyoto, Japan) was used as the light source. As shown in Fig 1B, the dorsal side of the finger was illuminated with light from the array, and the light transmitted through the finger was received by an IR-sensitive CCD camera (XC-EI50, Sony, Tokyo, Japan) with an IR-pass filter. As the result, the finger-vein pattern on the palm side of the finger was acquired. This method enables us to visualize the vein of which diameter is longer than about 1 mm. We cannot acquire a pattern of the capillaries which would be affected by smoking because of light scattering in the finger. A 640 x 480 pixel gray-scale still image was captured by a video-capture card (Monster TV Pocket, SKnet, Yokohama, Japan) on a notebook PC (PC-MP70G, Sharp, Osaka, Japan) and processed. A sample of finger image is shown as Fig 1C and its processed image is shown as Fig 1D. The LED current was adjusted manually whenever the intensity of the light transmitted through the finger was too high or low, and the angle of the palm to the vertical was maintained at less than several degrees when an image was captured.

Vein patterns of eight fingers (all except the thumbs) were acquired in two finger-vein images of each finger. The second image was obtained after taking the finger off the apparatus and setting the finger again. This apparatus was a prototype designed for acquiring each four images of both hands. For this purpose, a stopper or a guide bar for setting a finger reproducibly was not placed on the apparatus.

Room temperature was controlled by air conditioner between 25 and 28°C through the year. Rigid control of the temperature was not done.

### Sampled groups

The finger-vein patterns of 68 patients who had connective tissue diseases were acquired at the Department of Rheumatology and Clinical Immunology, Kyoto University Hospital in June and August 2006 and thereafter at three-month intervals through May 2007. The 68 patients included 26 with systemic sclerosis (SSc), 17 with systemic lupus erythematosus (SLE), 11 with primary Sjögren’s syndrome (SS), 10 with mixed connective tissue disease (MCTD), 2 with rheumatoid arthritis (RA) and 2 with dermatomyositis or polymyositis (DM/PM).

SSc, SLE and RA were diagnosed according to the criteria of the American College of Rheumatology [24–26], SS and MCTD according to the criteria of the Japanese Ministry of Health, Labour and Welfare [27, 28], and DM/PM according to Bohan and Peter’s criteria [29]. In the study period, new administration or change of medications, which affect the peripheral circulation of patients, were avoided.

The finger-vein patterns of 24 volunteers who did not have obvious diseases and did not take any medicine were acquired as references at the Hitachi Central Research Laboratory in June and August 2008 and thereafter at three-month intervals through May 2009. Sixty eight patients and 24 volunteers are all Japanese.

### Ethics statement

This study was designed in accordance with the Declaration of Helsinki and was approved by the ethics committees of both Graduate School of Medicine and Faculty of Medicine, Kyoto University and Hitachi Ltd. Written informed consent was obtained from patients and volunteers. Acquired data were stored on an anonymous basis and in a locked cabinet.

### Change of finger-vein patterns during one year and difference of patterns between patients and healthy volunteers

Change of finger-vein patterns themselves with time or season was checked by visual observation.

The index value of cross correlation between two images was used for personal identification. The method of image processing used to calculate this value has been reported elsewhere [19] and is described below. The time course of this value was used to detect whether or not there is a difference in the ability of personal identification between patients’ finger-vein patterns and healthy volunteers’ ones.

### Scheme of image processing and authentication

Fig 1 shows an example of an image of a finger-vein pattern captured with the CCD camera (C) and shows the enhanced vein pattern (D). The steps of the image processing (see S1 Fig) are described elsewhere [19]. The similarity between two registered images was evaluated on the basis of the coefficient of the correlation between them. Let *p* and *q* be *N* x *N* square matrices of image data; then the correlation between them is defined as

$$y_{i,j}=IFFT2\left[*\;FFT2\left(p\right)○FFT2\left(q\right)\right],\;i,j\;=\;1,2,\ldots \;,N,$$ (1)

where FFT2 denotes the two-dimensional fast Fourier transform (FFT), IFFT2 denotes the two-dimensional inverse FFT, * denotes the complex conjugate, and ○ denotes element-by-element multiplication. It was normalized as

$$Y_{i,j}=y_{i,j}/{\left[\sum_{k=1}^{N}\sum_{l=1}^{N}P_{k,l}{\left(u\right)}^{2}\;\cdot \;\sum_{k=1}^{N}\sum_{l=1}^{N}Q_{k,l}{\left(u\right)}^{2}\right]}^{1/2}\;,\;i,j,k,l=\;1,2,\;\ldots \;,N,$$ (2)

where *P*_*k*,*l*_(*u*) = * FFT2 (*p)*, *Q*_*k*,*l*_(*u)* = FFT2 (*q*).

The normalized cross correlation *C* between two images (*C* value) was defined as follows:

$$C={\left[\text{max}(Y_{i,j})\right]}^{1/2}.$$ (3)

When *C* = 1, the two images are identical. The size of an image captured by the CCD camera was 640 x 480 pixels. At first, an image with 512 x 480 pixels was cut out from it, and zero padding made an image with 512 x 512 pixels. The size of images used for authentication was 128 x 128 pixels. This size was optimized according to the method described elsewhere [19].

### Statistical analysis

Nonparametric methods, which do not assume a normal population distribution, were used for testing equality of population medians among groups. The Kruskal–Wallis test was used to test more than three groups’ data and Wilcoxon rank-sum test was used to test two groups’ data.

The Wilcoxon rank-sum test is a nonparametric alternative to the two-sample t-test, the basic steps of which are described below. The two sample sets are combined into one and ranked. The sum of the ranks, T, for either of the original sample sets is computed and compared with the critical value. If T is less than the critical value, there is no significant difference between the two groups’ data.

Multiple comparisons were performed to test which combinations of two groups among over three groups differed significantly, and the Bonferroni method was used to counteract the problem of multiple comparisons. There are two ways in the Bonferroni method. One is to adjust the significance level *α*, and the other is to adjust the significance probability p. When we test *n* independent hypotheses among over three groups, *α*(*n*) and p(*n*) should be adjusted as follows:

α(i)=α/n,i=1,2,3,…,n (4)

p(i)=p×n,i=1,2,3,…,n (5)

There is a statistical limitation in that the bigger the sample size, the smaller the p value. When dealing with large sample sizes, we can easily get statistically significant results whose effects are so small that they are of little or no importance [30]. We therefore use the effect size of the test result to know the real difference without the effect of sample size. The effect size *r* in the Wilcoxon rank-sum test is given by

$$r=\text{Z}/\sqrt{N},$$ (6)

where Z is the Z score and *N* is the total sample size, and the standard values of *r* for small, medium, and large effects are respectively 0.1, 0.3, and 0.5 [31]. Significant differences shown in the figures of this paper are ones for which the *r* is not lower than 0.3, which means that the effects are of at least medium size.

## Results

### Authentication results of patients and controls

Demographic features of patients and controls are listed in Table 1. As expected, the frequency of Raynaud’s phenomenon was higher in patients with SSc and MCTD than it was in patients with other diseases.

**Table 1:** Characteristics of patients and controls.

	SSc	SLE	SS	MCTD	RA	DM/PM	Healthy volunteers
Male/ Female	2/24	0/17	0/11	0/10	0/2	1/1	10/14
Age (mean±SD)	59.8±10.3	37.3±9.73	63.7±9.97	45.4±17.0	50.5±3.50	62.0±5.00	37.0±7.04
Frequency of Raynaud’s phenomenon	25/26 (96%)	4/17 (24%)	1/11 (9%)	10/10 (100%)	0/2 (0%)	0/2 (0%)	

SSc: systemic sclerosis, SLE: systemic lupus erythematosus, SS: Sjögren’s syndrome, MCTD: mixed connective tissue disease, RA: rheumatoid arthritis, DM: dermatomyositis, PM: polymyositis

**Fig 2:**
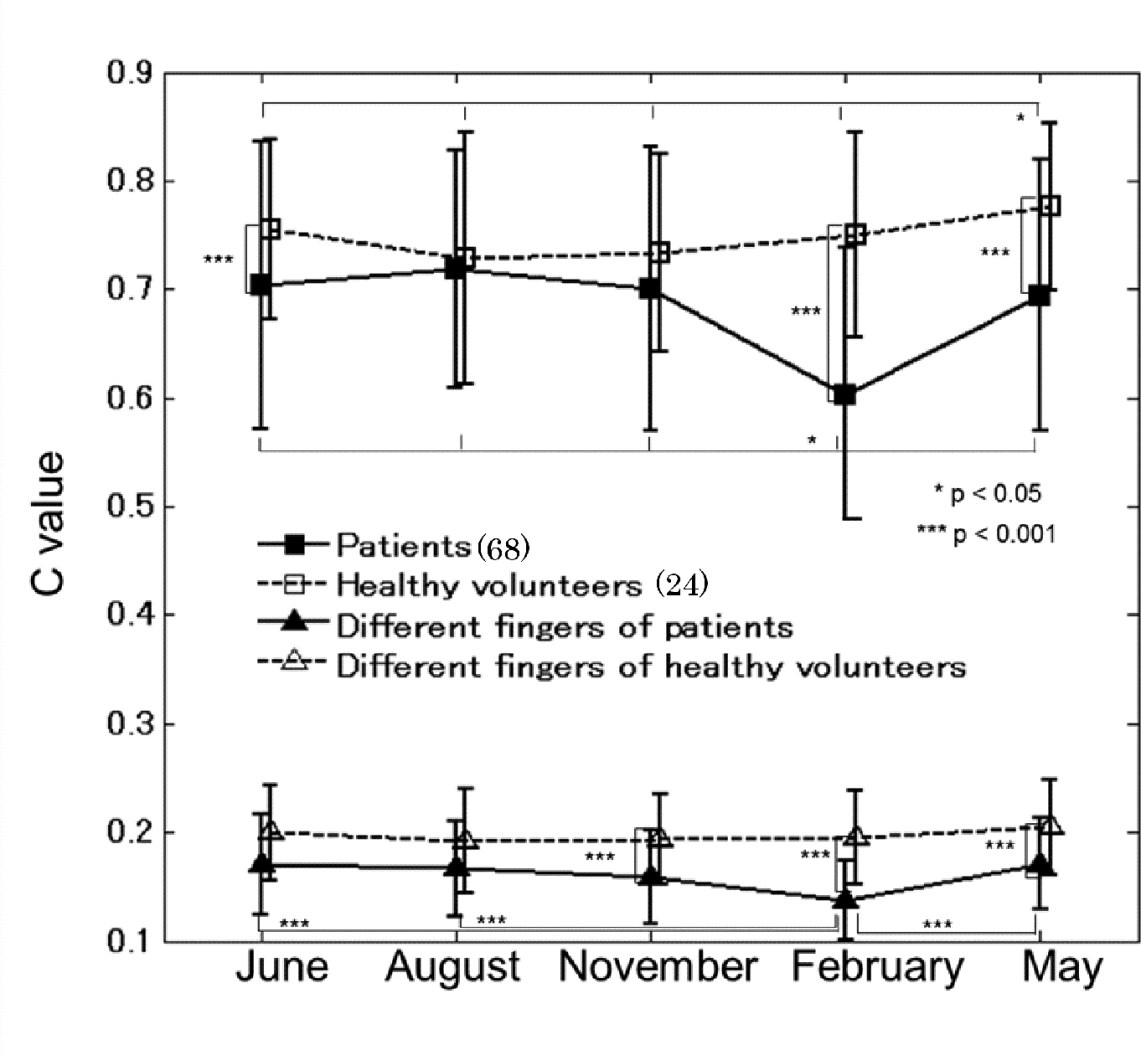
Same-finger and different-fingers authentication results for patients and healthy volunteers. Images obtained on the same day were used for both same-finger (⬛/⬜) and different-fingers (▲/△) authentication. Mean values plus/minus one standard deviation are plotted. *** p<0.001.

**Fig 3:**
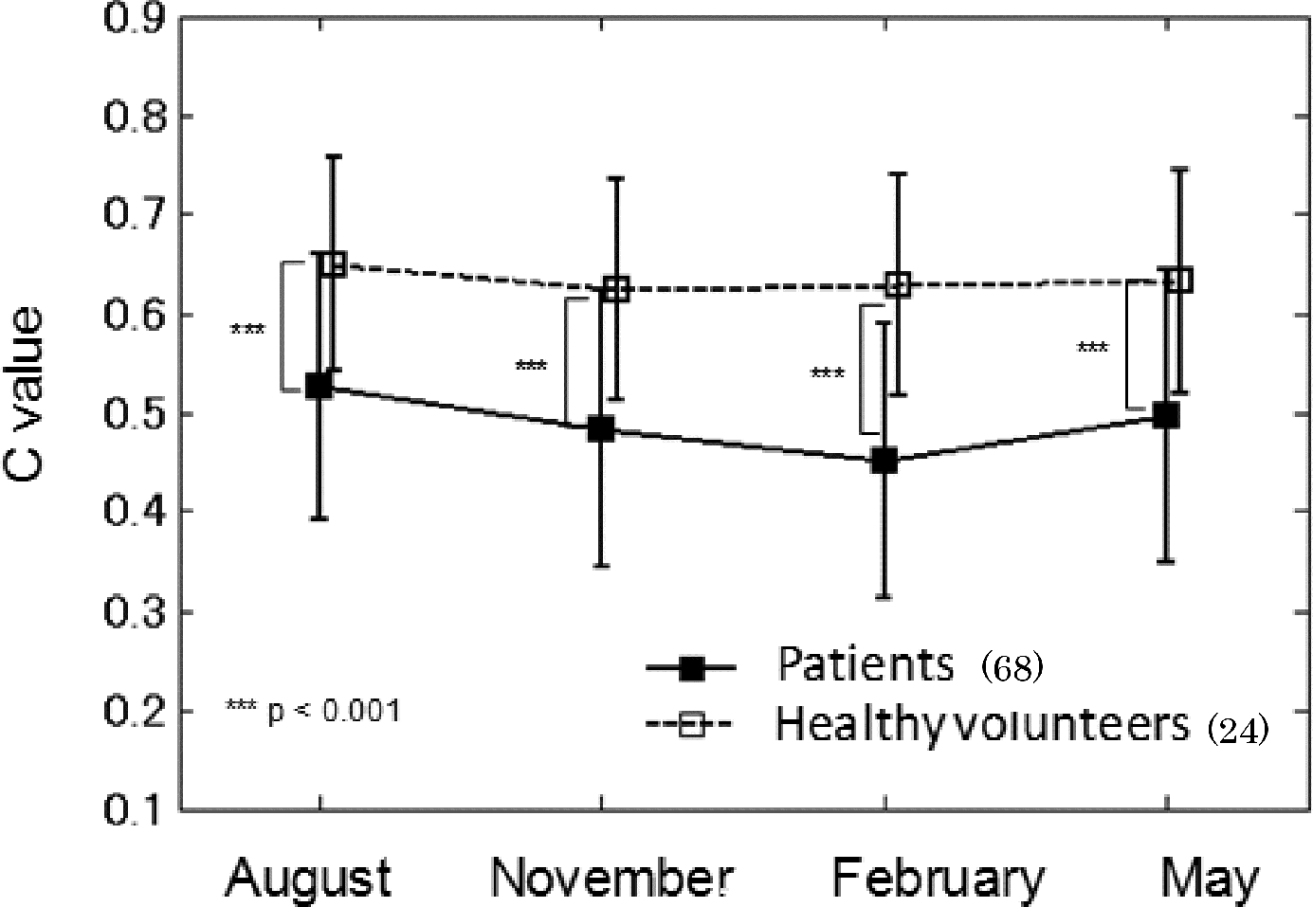
Time course of same-finger *C* values for images compared with those first acquired in June. Mean values plus/minus one standard deviation are plotted. *** p<0.001.

Fig 2 shows the same-finger and different-fingers authentication results for patients and healthy volunteers at each time of data acquisition. They are not the time-course data shown in Fig 3 but are instead the authentication results at each time of data acquisition, a total of five times during one year. Two images captured on the same day were used for authentication of both the same finger and different fingers. When the same-finger *C* values are separated perfectly from the different-fingers *C* values, we can identify one finger using uniform threshold.

Compared with the healthy volunteers’ same-finger *C* values, those of the patients showed significant (p<0.001) reductions in June, February, and May. And the different-fingers *C* values differed significantly (p<0.001) between patients and healthy volunteers in November, February, and May.

In patients, both same-finger and different-fingers’ authentication results showed significant (p<0.001) reductions of mean *C* values in February, whereas in healthy volunteers the authentication results in February showed no statistically significant reduction.

However *C* values of the same-finger are separated from those of the different-fingers both with patients and healthy volunteers. This result showed that there is no particular difficulty in authentication for patients as well as healthy volunteers.

Fig 3 shows the time course of same-finger *C* values obtained during one year when finger-vein images compared with those captured for the first time in June. Statistical analysis using the Wilcoxon rank-sum test with the effect size criteria described above revealed significant (p<0.001) differences between the authentication results of healthy volunteers and patients throughout the year.

The patients’ mean *C* values tended to be lower in winter, especially in February, but the results of Kruskal-Wallis tests and multiple comparisons with Bonferroni correction showed no statistically important differences between results obtained in different seasons.

### Authentication results based on types of diseases

To investigate which types of diseases contribute to the reduction of authentication results, we analyzed the results on a type-of-disease basis.

**Fig 4:**
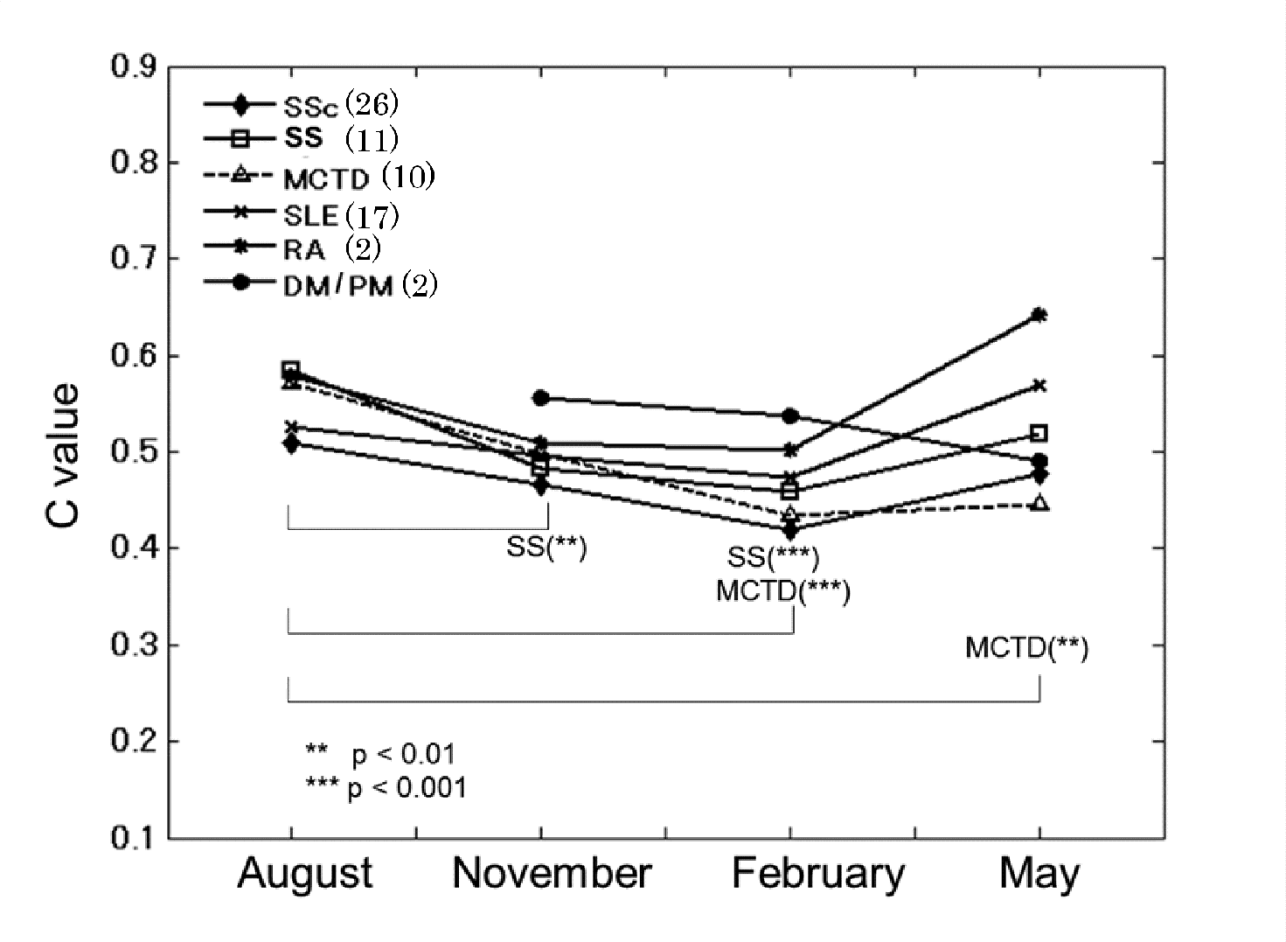
Time course, in each disease, of same-finger *C* values for images compared with those first acquired data in June. August DM/PM data could not be acquired because of the absence of patients. Mean values are plotted. ** p<0.01, *** p<0.001.

Fig 4 shows the time course of authentication results for patients classified by disease. For all diseases except DM/PM the *C* values tended to decline in February. The most profound reductions of mean *C* values in February were those for SSc and MCTD, in which most patients had Raynaud’s phenomenon.

The mean *C* values for SS patients were significantly (p<0.001) lower in November and February than they were in August, those for MCTD patients were significantly below the August level in both February (p<0.001) and May (p<0.01), and those for SLE patients differed significantly (p<0.001) between February and May.

The mean *C* values for SSc patients showed no significant difference(as shown in S1 Table), according to the criteria, effect size of 0.3 was used as a threshold, however the values are the lowest or second from the bottom compared with those for other diseases’ patients August through May. Furthermore the number of SSc patients is more than twice as much as that of other diseases’ patients. These results showed that SSc totally made a largest contribution in reduction of *C* values throughout the year.

## Discussion

We have examined how finger-vein pattern authentication is affected by the connective tissue diseases, especially SSc and MCTD because they cause defective circulation in fingers.

The results showed that the authentication of patients is more sensitive to seasonal change than that of healthy volunteers. In midwinter, February in Japan, the same-finger *C* values for patients are significantly lower than those for healthy volunteers. Thinning vein patterns in images captured in fall and winter were observed in this study, sometimes even in persons without apparent disease.

The apparatus used for the experiments was a prototype for acquiring each four fingers’ images of both hands. Greater the flexibility when a finger is set on the apparatus, lower the reproducibility. This allowed that *C* values even in healthy controls and in the same day-experiments was just around 0.7. On the other hand, false acceptance rate and false rejection rate of the commercial product of finger vein authentication technology are 0.0001% and 0.001% respectively [23].

We defined that there is a significant difference when effect size r is not lower than 0.3. According to this criteria, a difference between August and May in SS and August and November in MCTD of S1 Table were judged not to be significant, although we feel these differences are not ignorable.

Reduction of blood flow decreases the amount of hemoglobin available to absorb light in blood vessels. As a result, the light scattering inside the finger becomes stronger than the light absorption there. Thus impairment of peripheral circulation affect the change of vein pattern. This makes it difficult to acquire highly homologous vein patterns in summer and winter. On the other hand, skin roughness due to dry conditions could occur on both patients’ and healthy volunteers’ hands in winter. Easy scattering of light on the rough surface of the epidermis reduces the amount of incident light penetrating into the finger. Skin sclerosis with patients also affects this. The images acquired under this condition contain finger surface patterns much more than finger-vein patterns. These images could also differ from the images acquired in summer. These phenomena would result in the rejection of one’s same fingers as a different finger in winter. These seasonal changes in finger and vein patterns were larger in patients than they were in controls. Practical solution not to fail in authentication in cold season is to keep the fingers warm and promote blood circulation. In one year, the seasonal change was almost reversible but further data is needed to investigate whether the change gradually progresses each year or not.

For most patients with connective tissue diseases there might be no particular problems in using our finger-vein pattern authentication system through the year. Some attention would be needed for the patients with SSc with severe skin sclerosis of fingers because fewer finger–vein patterns were observed with patients with SSc. Reduction of blood flow, dermal sclerosis, and skin roughness affect the vein pattern image and reduce the *C* values of patients with SSc. There is also a possibility that false rejection may occur using a uniform threshold, and further studies on these points might be needed. As for vascular abnormalities in the hands, Allanore Y. et al. reported that SSc patients showed abnormalities of both arterial and venous of small caliber as well as the microcirculation [32]. In this literature, abnormalities such as a digital artery that did not reach the first phalanx and a lack of visible venous return were shown.

Raynaud’s phenomenon might make authentication difficult, but we did not observe Raynaud’s phenomenon during the year that the finger-vein patterns of the patients were obtained. One reason is that even in winter it was not so cold inside the hospital.

This method is noninvasive and only a few seconds are needed to capture finger images and calculate *C* values. Changes of *C* values reflects the change of blood circulation totally, thus seasonal changes of patients’ *C* values indicated that this method could be used to objectively evaluate the severity of impairment in peripheral circulation of SSc, SS, and MCTD patients. Although this method may not contribute to the precision of classification of connective tissue diseases, it has a possibility to assist evaluation of the severity of impairment in peripheral circulation noninvasively.

Furthermore we have observed phenomena that distal joints with patients transmit near-infrared light more easily than proximal ones. This indicates that impairment of blood flow start from terminal. Though further data and validation are required, quantification of this phenomenon has some potential for clinical application, for example, change index of impairment in peripheral circulation.

This finger-vein pattern authentication method is therefore potentially able to improve the diagnosis of connective tissue diseases. Expansion of used wavelength range of light has another potential of getting other information about the state of fingers than blood circulation.

## Supporting Information

S1 Fig Scheme of image processing for authentication. (DOC)

S1 Table Multiple comparisons by Wilcoxon rank-sum test on SSc, SS, MCTD, and SLE patients’ *C* values data. (DOC)

## References

[1] Trauring M. Automatic comparison of finger-ridge patterns. Nature. 1963; 197: 938–940. 13994002 10.1038/197938a0

[2] Jain A, Hong L, Pankanti S, Bolle R. An identity authentication system using fingerprints. Proceedings of the IEEE. 1997; 85(9): 1365–88.

[3] Tico M, Kuosmanen P, Saarinen J. Wavelet domain features for fingerprint recognition. Electronics Letters. 2001; 37(1): 21–2.

[4] Maltoni D, Maio D, Jain AK, Prabhakar S. Handbook of Fingerprint Recognition. 2nd ed. Springer; 2009.

[5] Daugman JG. High confidence visual recognition of persons by a test of statistical independence. IEEE Trans. Pattern Recognition and Machine Intelligence. 1993; 15(11): 1148–61.

[6] Daugman JG. Mathematical Explanation of Iris Technologies. http://www.cl.cam.ac.uk/~jgd1000/math.html. [accessed Feb 5, 2015].

[7] Bledsoe WW. The model method in facial recognition Panoramic research Inc., Palo Alto, CA, Rep. PRI: 15, 8 1966.

[8] Kanade T. Picture processing system by computer complex and recognition of human faces. Technical Report Dept. of Information Science, Kyoto University 11 1973.

[9] Turk MA, Pentland AP. Face recognition using eigenfaces. Proceedings of IEEE Conference on Computer Vision and Pattern Recognition. Institute of Electrical and Electronics Engineers; New York; 1991: 586–91.

[10] Sidlauskas DP. 3D hand profile identification apparatus. US Patent. 1988: 4,736,203.

[11] Jain AK, Ross A, Pankanti S. A prototype Hand Geometry-based Verification System. Proceedings of 2nd Int’l Conference on Audio- and Video-based Biometric Person Authentication (AVBPA); 1999 March 22–24; Washington D.C.; 1999: 166–71.

[12] Im SK, Park HM, Kim YW, Han SC, Kim SW, Kang CH. An Biometric Identification System by Extracting Hand Vein Patterns. J Korean Physical Society. 2001; 38(3): 268–72.

[13] Im SK, Choi HS, Kim SW. A Direction-Based Vascular Pattern Extraction Algorithm for Hand Vascular Pattern Verification. ETRI Journal. 2003; 25(2): 101–8.

[14] International Organization for Standardization. JTC 1/ SC37 Technical Committees of Biometrics. http://www.iso.org/iso/jtc1_sc37_home [accessed Feb 5, 2015].

[15] Hardy JD, Hammel HT, Murgatroyd D. Spectral transmittance and reflectance of excised human skin. J Appl Physiol. 1956; 9: 257–64. 13376438 10.1152/jappl.1956.9.2.257

[16] Wilson BC, Patterson MS, Flock ST, Moulton JD. The optical absorption and scattering properties of tissues in the visible and near-infrared wavelength range In: Douglas RH, Moan J, and Acqua FD, editors. Light in Biology and Medicine; Vol. 1, New York: Plenum Press; 1988: 45–52.

[17] Key H, Jackson PC, Wells PNT. New approaches to transillumination imaging. J Biomed Eng. 1998; 10: 113–8. 10.1016/0141-5425(88)90084-2 3361865

[18] Shimizu K. [Optical trans-body imaging: feasibility of optical CT and functional imaging of living body]. Japanese J. of medicina philosophica. 1992; 11(8): 620–629. Japanese.

[19] Kono M, Ueki H, Umemura S. Near-infrared finger vein patterns for personal identification. Applied Optics. 2002; 41(35):7429–36. 12502300 10.1364/ao.41.007429

[20] Miura N, Nagasaka A, Miyatake T. Feature extraction of finger-vein patterns based on repeated line tracking and its application to personal identification. Machine Vision and Applications. 2004; 15 (4): 194–203.

[21] Miura N, Nagasaka A, Miyatake T. Extraction of Finger-Vein Patterns Using Maximum Curvature Points in Image Profiles. IEICE—Transactions on Information and Systems. 2007; E90-D(8): 1185–94.

[22] Vein ID, http://www.hitachi.co.jp/products/it/veinid/global/index.html, [accessed Aug 26, 2015].

[23] Hitachi Finger Vein H1 Unit, http://www.hitachi.eu/veinid/documents/h1brochurelatest.pdf, [accessed Aug 26, 2015].

[24] Preliminary criteria for the classification of systemic sclerosis (scleroderma). Subcommittee for scleroderma criteria of the American Rheumatism Association Diagnostic and Therapeutic Criteria Committee. Arthritis Rheum 1980; 23: 581–590. 7378088 10.1002/art.1780230510

[25] Hochberg MC. Updating the American College of Rheumatology revised criteria for the classification of systemic lupus erythematosus. Arthritis Rheum. 1997;40:1725. 10.1002/art.1780400928 9324032

[26] Arnett FC, Edworthy SM, Bloch DA, McShane DJ, Fries JF, et al: The American Rheumatism Association 1987 revised criteria for the classification of rheumatoid arthritis. Arthritis Rheum. 1988; 31: 315–324. 3358796 10.1002/art.1780310302

[27] Fujibayashi T, Sugai S, Miyasaka N, Hayashi Y, Tsubota K. Revised Japanese criteria for Sjögren’s syndrome (1999): availability and validity. Mod Rheumatol. 2004; 14: 425–434. doi: 10.3109/s10165-004-0338-x 24387718 10.3109/s10165-004-0338-x

[28] Kasukawa R, Tojo T, Miyawaki S, Yoshida H, Tanimoto K, Nobunaga M, et al Preliminary diagnostic criteria for classification of mixed connective tissue disease In: Kasukawa R, Sharp GC, editors. Mixed connective tissue disease and antinuclear antibodies. Amsterdam: Excerpta Medica; 1987: 41–47.

[29] Bohan A, Peter JB: Polymyositis and dermatomyositis (first of two parts). N Engl J Med. 1975; 292: 344–347. 1090839 10.1056/NEJM197502132920706

[30] Null hypothesis testing and effect sizes, http://staff.bath.ac.uk/pssiw/stats2/page2/page14/page14.html [accessed Aug 26, 2015]

[31] Cohen J. The Effect Size: r In: Statistical power analysis for the behavioral sciences. 2nd ed. Hillsdale: Lawrence Erlbaum; 1988: 2146–86.

[32] Allanore Y, Seror R, Chevrot A, Kahan A, Drapé JL. Hand vascular involvement assessed by magnetic resonance angiography in systemic sclerosis. Arthritis Rheum. 2007; 56(8):2747–54. 17665441 10.1002/art.22734

