# Supplementary figures and images for: Personal Authentication Analysis Using Finger-Vein Patterns in Patients with Connective Tissue Diseases—Possible Association with Vascular Disease and Seasonal Change -
Source: PLoS One. 2015 Dec 23;10(12):e0144952. doi: 10.1371/journal.pone.0144952 (PMC4689452; doi:10.1371/journal.pone.0144952)

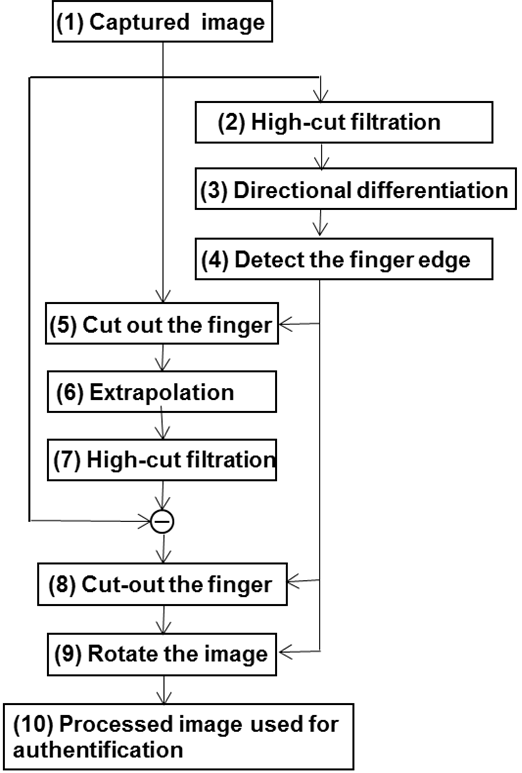


S1 Fig

Supplement: S1 Fig — (DOC) [file pone.0144952.s001.doc]
