# Supplementary material for: Personal Authentication Analysis Using Finger-Vein Patterns in Patients with Connective Tissue Diseases—Possible Association with Vascular Disease and Seasonal Change -
Source: PLoS One. 2015 Dec 23;10(12):e0144952. doi: 10.1371/journal.pone.0144952 (PMC4689452; doi:10.1371/journal.pone.0144952)
Supplement: S1 Table — (DOC) [file pone.0144952.s002.doc]

**S1 Table. Multiple comparisons by Wilcoxon rank-sum test on SSc, SS, MCTD, and SLE patients’ *C* values data.**

|  | Data set of *C* values | P value | Effect size (*r*) |
| --- | --- | --- | --- |
| SSc | August and November | 0.108 | 0.139 |
|  | August and February | 4.20 x10^-5^(***) | 0.268 |
|  | August and May | 0.679 | 0.0961 |
|  | November and February | 0.156 | 0.137 |
|  | November and May | 1.00 | 0.0413 |
|  | February and May | 0.0546 | 0.167 |
| SS | August and November | 2.79 x10^-4^ (***) | 0.343 (^‡^) |
|  | August and February | 6.21 x10^-6^ (***) | 0.339 (^‡^) |
|  | August and May | 0.0336 (*) | 0.224 |
|  | November and February | 1.00 | 0.0720 |
|  | November and May | 0.734 | 0.122 |
|  | February and May | 0.0588 | 0.199 |
| MCTD | August and November | 0.0438 (*) | 0.274 |
|  | August and February | 8.00 x10^-4^ (***) | 0.478 (^‡^) |
|  | August and May | 1.31 x10^-3^ (**) | 0.413 (^‡^) |
|  | November and February | 0.272 | 0.204 |
|  | November and May | 1.00 | 0.125 |
|  | February and May | 1.00 | 0.0642 |
| SLE | August and November | 1.00 | 0.102 |
|  | August and February | 0.0744 | 0.213 |
|  | August and May | 0.479 | 0.160 |
|  | November and February | 1.00 | 0.110 |
|  | November and May | 0.0852 | 0.235 |
|  | February and May | 8.69 x10^-4^ (***) | 0.385 (^‡^) |

* p < 0.05, ** p < 0.01, *** p < 0.001, ^‡^ *r* ≥ 0.3

P value was corrected by Bonferroni method.

All data sets of *C* values were calculated based on first acquired data in June.
